# Closed‐loop auditory stimulation of slow‐wave sleep in chronic insomnia: a pilot study

**DOI:** 10.1111/jsr.14179

**Published:** 2024-03-11

**Authors:** Daniela Dudysová, Karolina Janků, Marek Piorecký, Veronika Hantáková, Mária Orendáčová, Václava Piorecká, Jan Štrobl, Monika Kliková, Hong‐Viet V. Ngo, Jana Kopřivová

**Affiliations:** ^1^ National Institute of Mental Health Klecany Czech Republic; ^2^ Third Faculty of Medicine Charles University Prague Czech Republic; ^3^ Faculty of Biomedical Engineering Czech Technical University in Prague Prague Czech Republic; ^4^ School of Medicine, Medical Sciences and Nutrition University of Aberdeen Aberdeen Scotland; ^5^ Department of Physiology, Faculty of Science Charles University in Prague Prague Czech Republic; ^6^ Center for Brain, Behaviour and Metabolism University of Lübeck Lübeck Germany; ^7^ Department of Psychology University of Lübeck Lübeck Germany; ^8^ Department of Psychology University of Essex Colchester UK

**Keywords:** closed‐loop auditory stimulation, insomnia, memory consolidation, polysomnography, slow oscillation

## Abstract

Insomnia is a prevalent and disabling condition whose treatment is not always effective. This pilot study explores the feasibility and effects of closed‐loop auditory stimulation (CLAS) as a potential non‐invasive intervention to improve sleep, its subjective quality, and memory consolidation in patients with insomnia. A total of 27 patients with chronic insomnia underwent a crossover, sham‐controlled study with 2 nights of either CLAS or sham stimulation. Polysomnography was used to record sleep parameters, while questionnaires and a word‐pair memory task were administered to assess subjective sleep quality and memory consolidation. The initial analyses included 17 patients who completed the study, met the inclusion criteria, and received CLAS. From those, 10 (58%) received only a small number of stimuli. In the remaining seven (41%) patients with sufficient CLAS, we evaluated the acute and whole‐night effect on sleep. CLAS led to a significant immediate increase in slow oscillation (0.5–1 Hz) amplitude and activity, and reduced delta (1–4 Hz) and sigma/sleep spindle (12–15 Hz) activity during slow‐wave sleep across the whole night. All these fundamental sleep rhythms are implicated in sleep‐dependent memory consolidation. Yet, CLAS did not change sleep‐dependent memory consolidation or sleep macrostructure characteristics, number of arousals, or subjective perception of sleep quality. Results showed CLAS to be feasible in patients with insomnia. However, a high variance in the efficacy of our automated stimulation approach suggests that further research is needed to optimise stimulation protocols to better unlock potential CLAS benefits for sleep structure and subjective sleep quality in such clinical settings.

## 1 INTRODUCTION

Insomnia is the second most common mental disorder in Europe (Wittchen et al., 2011) affecting ~10% of the population (Ohayon, 2002). Insomnia is a disabling condition increasing the risk of cognitive impairments, mood disturbances, and the development of mental disorders, such as depression (Baglioni et al., 2011). Although a majority of patients may significantly benefit from cognitive behavioural therapy, first‐line treatment (Riemann et al., 2017) or pharmacotherapy, there is still a substantial proportion of patients who do not respond or respond sufficiently to these standard treatments (Murtagh & Greenwood, 1995; Okajima et al., 2011). Moreover, some therapeutic interventions, such as sleep restriction, may be demanding or associated with negative side‐effects (Kyle et al., 2014). Likewise, pharmacotherapy can also pose similar difficulties (Atkin et al., 2018). Thus, it is essential to explore potential new therapeutic approaches that could be used as alternative or complementary interventions to the established insomnia treatments.

Although the diagnosis of insomnia is based on subjective complaints, higher sleep fragmentation, reduced slow‐wave sleep (SWS), and reduced rapid eye movement (REM) sleep have been consistently described by polysomnography (PSG) in patients with insomnia (Baglioni et al., 2014). Of note, both SWS and REM sleep stages significantly contribute to memory processing and their disruption may thus have a further impact on the daytime functioning of patients with insomnia. Indeed, deficits in overnight consolidation of hippocampal‐dependent declarative memory (Backhaus et al., 2006; Nissen et al., 2011), procedural memory (Cellini et al., 2014), as well as deficits in sleep‐dependent emotional processing (Wassing et al., 2019) have been reported in this clinical group. Patients with insomnia also show persistent cortical activation related to the autonomic and central nervous system hyperarousal (Riemann et al., 2010), which is reflected by increased activity in faster electroencephalography (EEG) frequencies, such as beta 1 (16–24 Hz), beta 2 (25–32 Hz), and reduced delta (1–4 Hz) activity (Svetnik et al., 2017).

Thus, non‐invasive brain stimulation methods to target specific sleep characteristics or to decrease arousal may represent a viable approach to improving the treatment of insomnia (Geiser et al., 2020). Research of the last decade showed that while phase‐locked auditory stimulation (closed‐loop auditory stimulation [CLAS]) does not affect the sleep macrostructure, it could intervene in specific sleep dynamics and promote sleep slow oscillations (SOs), sleep spindles, and memory consolidation (Ngo et al., 2013; Papalambros et al., 2017; Stanyer et al., 2022). Moreover, CLAS may be used as means to decrease sensitivity to outside noise and thus protect sleep (Pathak et al., 2021). Thus, it is highly intriguing to test whether this entirely non‐invasive approach could improve sleep in clinical conditions such as insomnia.

Building on the knowledge gaps, the present study aimed to assess the feasibility of CLAS in insomnia and its effect on sleep macro‐ and microstructure. We hypothesised there would be no alterations of sleep macrostructure after CLAS, but that the CLAS would enhance the SOs and SWS activity as well as the duration, density, and amplitude of sleep spindles. These changes were expected to relate to the improvement in a declarative memory task and increased subjective sleep quality. As a part of the exploratory analysis, we aimed to assess changes in alpha and beta EEG bands to see the possible effect of CLAS on arousal during sleep.

## 2 METHODS

### 2.1 Participants

A total of 27 patients with chronic insomnia were recruited at the Department of Sleep Medicine of the National Institute of Mental Health, Czech Republic (NIMH‐CZ), and via online advertisement. Inclusion criteria were: (a) diagnosis of chronic insomnia based on *International Classification of Sleep Disorders*, third edition criteria, (b) complaints about problems with maintaining sleep, and (c) aged ≥18 years. The exclusion criteria were: (a) medication affecting sleep, (b) severe psychiatric, neurological, or somatic comorbidity, (c) usage of cognitive stimulants, such as caffeine, tobacco, and energy drinks 6 h before the experiment, and (d) extreme chronotype. The study was approved by the Ethics Committee of the NIMH‐CZ, and all participants signed informed consent before participation.

### 2.2 Study design and procedure

A within‐subject, randomised, crossover, sham‐controlled study design was conducted. After completing a screening questionnaire on inclusion and exclusion criteria, the patients spent 2 experimental nights at the NIMH‐CZ sleep laboratory, separated by at least 7 days. During each night, the patients received either stimulation (CLAS) or sham stimulation. The order of the stimulation versus sham condition was randomised and balanced across patients. The patients were blinded to the assigned condition at the time of the experiment. During the sham condition, the same EEG tracing procedure was followed as in the stimulation condition. However, no sound was delivered to the headphones. The patients were instructed to refrain from napping as well as from alcohol, caffeine, and drugs on the day of the experiments.

Before and after each experimental night, the patients completed the sleep quality scales and questionnaires and completed a word‐pair memory task (Dudysová et al., 2016) adapted from Ngo et al. (2013). Lights were turned off at 10:00 p.m., varying slightly due to individual differences, such as sleep time preparation. Each patient was given a sleep opportunity of 8 h, during which PSG was recorded, thus they were awakened at ~6:00 a.m.

### 2.3 Polysomnography

A whole‐night PSG was recorded. All recordings included EEG according to the 10/20 standard system, electro‐oculography, electromyography (three submental electrodes), electrocardiography, and video monitoring. The EEG included seven channels (Fpz, F3, F4, C3, C4, P3, and P4) referenced to mastoids (M1 and M2) on the contralateral side of the scalp. Data were recorded using the Brainscope PSG system (M&I spol. s.r.o., Czech Republic) with a band‐pass filter of 0.1–200 Hz and a sampling rate of 1 kHz. Two independent raters visually scored all the records according to the American Academy of Sleep Medicine criteria (AASM, 2007). The ‘EEG Viewer’ software, version 2019 (Unimedis s.r.o., Czech Republic) was used for scoring and calculation of sleep macrostructure parameters, including duration (min) and proportion (percentage) of total sleep time (TST), time of wakefulness, number of arousals, and time spent in all sleep stages (NREM 1, NREM 2, NREM 3, REM) for each experimental night. Arousals were computed for both conditions. All arousals with a duration of 3–15 s throughout the night were marked, and the arousal index (*n*/h) was computed for each condition.

A statistical analysis of the sleep macrostructure was performed via the Wilcoxon signed‐rank test with false discovery rate (FDR) correction for multiple comparisons. For all statistical analyses, we report means, standard deviations (SDs), and effect sizes (r) where appropriate. All analyses were done using the IBM Statistical Package for the Social Sciences (SPSS®; IBM Corp., Armonk, NY, USA) and MATLAB software (MathWorks Inc., Natick, MA, USA).

### 2.4 Closed‐loop auditory stimulation

Following the original methodology of Ngo et al. (2013), acoustic stimulation was presented during the first two sleep cycles in SWS. Once the SWS stage was visually detected by the experimenter and stable for at least 3 min, the stimulation protocol was initiated by the researcher. In case of a movement, awakening, or a shift to a different sleep stage, the stimulation was manually switched off by the investigator until the next SWS was reached again. Python application was used for the online detection of SOs from the averaged signal at F3 and F4 electrodes. The threshold for SOs detection was set to −80 μV for participants aged ≤30 years (Ngo et al., 2013) and to −40 μV for participants who were >30 years (Papalambros et al., 2017) to provide sufficient stimulation across all ages. The CLAS was employed in phase with SO up‐states (Ngo et al., 2013). Once the negative half‐wave peak of SO was detected, two successive pink noise pulses (1/f), each 50 ms long, were delivered. Pink noise was used instead of white noise as it is softer and more comfortable for hearing and was previously widely used in sleep studies as an auditory stimulus (Ngo et al., 2013; Papalambros et al., 2017). The first pulse occurred during the predicted up‐phase of the detected SO and the second pulse followed 1.075 s after the first pulse. These two pulses were followed by a 2.5 s pause before the detection process resumed. In the sham condition, the detection process was the same, but no sounds were delivered. The time points of the predicted stimulations (sham stimuli) were marked in the PSG recording for subsequent analysis. The auditory stimuli were delivered binaurally via soft all‐rubber headphones, suitable for sleep (Maxrock, model: EL‐273707). The sound intensity was set before every experimental night subjectively by each patient to a level that was not disturbing but still detectable.

### 2.5 Acute effect of CLAS

All analyses were performed with MATLAB, R2019a software (MathWorks Inc.). An identical analysis was used for the stimulation and sham recordings. To ensure CLAS accuracy, a phase analysis was performed using the MATLAB toolbox ‘FieldTrip’ (Oostenveld et al., 2011). Data were filtered by a finite impulse response (FIR) bandpass filter in the range 0.5–4.0 Hz; a demean filter was applied as well. The mean of the F3 and F4 electrodes was used for the consequent analysis. The outliers were defined on the segments/trials level, where the whole segments were defined as outliers when they were more than three interquartile amplitude ranges above the upper quartile (75%) or below the lower quartile (25%). The 4 s‐long segments were extracted (1 s before and 3 s after the first stimulation), and the Hilbert transformation was used for phase values extraction. The phase values were computed for the first and second stimulation pulses during both sham and stimulation conditions. The evaluation was performed by the pair‐wise non‐parametric Wilcoxon test. The ‘CircStat’ toolbox (Berens, 2009) for MATLAB was used for the descriptive statistical evaluation of the phase targeting.

Relative spectral powers were calculated as the ratio of the contribution of individual bands, that is, the power of a given band divided by the total power (0.5–30 Hz). The analysis of the relative power concerning the effect of CLAS followed an identical procedure. Data were filtered in the range of 0.5–30.0 Hz, and the demean filter was applied. The mean of F3 and F4 electrodes was used for the consequent analysis. The outliers were again defined on the segments/trials level, where the whole segments were defined as outliers when they are more than three interquartile ranges above the upper quartile or below the lower quartile. No subsequent visual inspection was performed within sleep segments, as we did not expect the occurrence of high frequency muscle activity to affect our examined frequency range. Relative power spectra analysis was performed for the 4 s‐long segments of ‘ON’ and ‘OFF’ intervals of EEG records, where the ON intervals originated 1 s before the detection and the OFF intervals started 1 s after the second stimulation. The multi‐taper method with a Hanning window was used for the power‐spectra estimation. The relative power spectra were computed for SOs (0.5–1.0 Hz) and delta bands (1.0–4.0 Hz).

The effect of stimulation was analysed according to the amplitude of EEG segments for both the stimulation and sham conditions. The segments were aligned around the stimulation/sham time‐point and the Fpz electrode was chosen for analysis. The amplitude and baseline corrected amplitude were analysed via the non‐parametric statistical test equipped with cluster‐based correction for multiple comparisons (Maris & Oostenveld, 2007). The baseline interval was chosen for a well‐synchronised detection period from 0.5 to 0.35 s before the first stimulus, for detail please see (Piorecky et al., 2021). Outlying data segments exceeding the amplitude 300 μV or segments with an amplitude below 10 μV were excluded.

### 2.6 The EEG power spectral analyses

All analyses were performed with MATLAB, R2019a software (MathWorks Inc.). An identical analysis was used for the stimulation and sham recordings. A visual artefact rejection, including movement and electrode artefacts, was done manually in 5‐s intervals. If a segment included an artefact, it was discarded regardless of whether the artefact affected only a part or the whole segment. First, power spectral analyses focused on all SWS episodes from the whole stimulation versus sham night. Frequency bands included in the EEG analysis were SOs (0.5–1.0 Hz), delta (1–4 Hz), and sigma (12–15 Hz) corresponding to the frequency band of fast sleep spindles. Both absolute and relative power spectra values were computed according to Mander et al. (2013). Relative power was again computed as a proportion of total power. Second, the analyses were conducted separately on the first two sleep cycles because CLAS was conducted during these sleep cycles due to the physiologically higher quantity of SWS in this part of the night. To see whether the CLAS enhanced or reduced faster EEG activity (as a sign of heightened arousal), we also focused on alpha (8–12 Hz), beta 1 (15–20 Hz), and beta 2 (20–30 Hz) bands. The analyses were performed for all electrodes (C3, C4, F3, F4, P3, and P4) for SWS. Then, statistical analysis was performed via the Wilcoxon signed‐rank test with FDR correction.

### 2.7 Sleep spindles analysis

To examine sleep spindles, the automatic detection of discrete sleep spindle events was adapted from previous studies (Ferrarelli et al., 2007), using a frequency band of 12–15 Hz. The analysis was performed on six channels (F3, F4, C3, C4, P3, and P4) for all SWS intervals during the night. Sleep spindles were investigated for density (*n*/min), the average duration of a single spindle (s), and power‐integrated spindle amplitude (μV/min).

### 2.8 Memory task

A Czech version of the word‐pair association task was used (Dudysová et al., 2016) to assess declarative memory performance on both visits. This method has been previously used in numerous studies assessing declarative memory and sleep (Marshall et al., 2004; Payne et al., 2012), as well as in studies using acoustic stimulation during sleep and investigating its effect on memory consolidation (Ngo et al., 2013; Papalambros et al., 2017). Two versions of the test were used for each patient and were presented in a randomised and balanced order. The task consisted of 120 moderately semantically related word pairs (e.g., apple‐peach, consciousness‐brain) in a randomised order for each test session. All 120‐word pairs were presented to the participant in the learning phase of the test on a computer screen for 4 s, with an inter‐stimulus interval of 1 s. The first word in the pair was a cue word, and the second word was required to be recalled later (target word). Participants were asked to remember as many pairs as possible. After the learning, a recall test with feedback followed. The same word pairs were presented but in different, random order. Following a 5‐min break, the same recall test, with word pairs in a different order, was completed by the patients, but no feedback was given.

The fourth and final task phase was done in the morning ≥30 min after the patients woke up. Memory consolidation was computed as the difference between the amount of correctly recalled pairs in the morning and evening, which is further divided by the amount of correctly recalled pairs in the evening and multiplied by 100. This relative memory consolidation value was then used for a comparison between the stimulation and sham conditions via the Wilcoxon signed‐ranks test.

### 2.9 Self‐reported scales and questionnaires

Questionnaires to assess different individual aspects of sleep were completed: the Epworth Sleepiness Scale (Johns, 1991) to evaluate a level of daytime sleepiness and the Morningness–Eveningness Questionnaire (Horne & Ostberg, 1976) to check for extreme chronotypes. Additionally, the Beck Depression Inventory‐2 (Beck et al., 1961) and Beck Anxiety Inventory (Beck et al., 1988) were used to control for the severity of subjectively experienced symptoms of depression and anxiety.

The patients completed a three‐part questionnaire to evaluate subjective sleep quality during the experimental nights. In the morning after the experiment, the patients evaluated how rested they felt on a 3‐point Likert scale (‘completely rested’, ‘partly rested’, ‘not rested at all’). For the second question, they had to rate the quality of their sleep during the experimental night on a 4‐point Likert scale (‘very bad’, ‘bad/superficial’, ‘pretty good’, ‘good/refreshing’). The last question asked how much sleep in hours participants perceived to get.

## 3 RESULTS

### 3.1 Feasibility of CLAS

As part of assessing the feasibility of CLAS in chronic insomnia, we evaluated the attrition rates and the overall amount of stimulation in our patients.

From a sample of 27 patients (mean [SD, range] age 36.6 [14.0, 20–59] years), four were excluded from the study because of an absence of acoustic stimuli due to insufficient opportunity for CLAS (four of 27 patients, i.e., 14.8%); three were excluded as a result of bad quality PSG recordings; one due to the presence of an anxiety disorder; and one because of previously undiscovered use of medication affecting sleep. Furthermore, one patient withdrew from the study due to the discomfort experienced during the first experimental night. Therefore, 17 patients were included in the analyses. The demographic and clinical characteristics of the final sample are presented in Table 1.

**TABLE 1:** Demographic and clinical characteristics of the whole included sample (17 patients) and a reduced sample with sufficient number of stimulations (7 patients).

Variable	Initial analysis (*n* = 17)	Sufficient stimulation (*n* = 7)
Age, years, mean (SD)	33.53 (13.99)	28.0 (13.86)
Sex, females/males, *n*	10/7	3/4
ESS score, mean (SD)	8.31 (4.03)	5.33 (2.21)
MEQ score, mean (SD)	55.31 (10.97)	51.83 (4.74)
BDI‐II score, mean (SD)	11.56 (7.57)	8.50 (3.55)
BAI score, mean (SD)	8.13 (5.63)	7.17 (3.80)

*Note*: mean (± SD) scores of questionnaires are presented.

Abbreviations: BAI, Beck Anxiety Inventory; BDI‐II, Beck Depression Inventory‐II; ESS, Epworth Sleepiness Scale; MEQ, Morningness–Eveningness Questionnaire.

The mean (range) number of stimulation cycles (including SO detection, first and second stimulation)/night was 125.88 (2–413), that is, a total of 4–826 stimulations/night. To evaluate the effect of CLAS in chronic insomnia, only patients with a sufficient number of stimulations were included in the analyses of sleep macro‐ and microstructure. A minimum of 50 stimuli was set as an appropriate criterion for unbiased and significant evaluation based on our previous analyses (Piorecky et al., 2021) and another published study (Debellemaniere et al., 2018), yielding a sample size of seven patient (four men, mean [SD, range] age 28 [13.86, 20–59] years) for the following analyses (Table 1). There was no significant difference in age between the patients excluded due to insufficient or no stimulation (14 patients; mean [SD] age 39.29 [12.73] years) and the reduced sample with sufficient stimulations (seven patients; mean [SD] age 28.0 [13.86] years; *Z* = −1.572; *p* = 0.128).

### 3.2 Acute effect of CLAS

**FIGURE 1:**
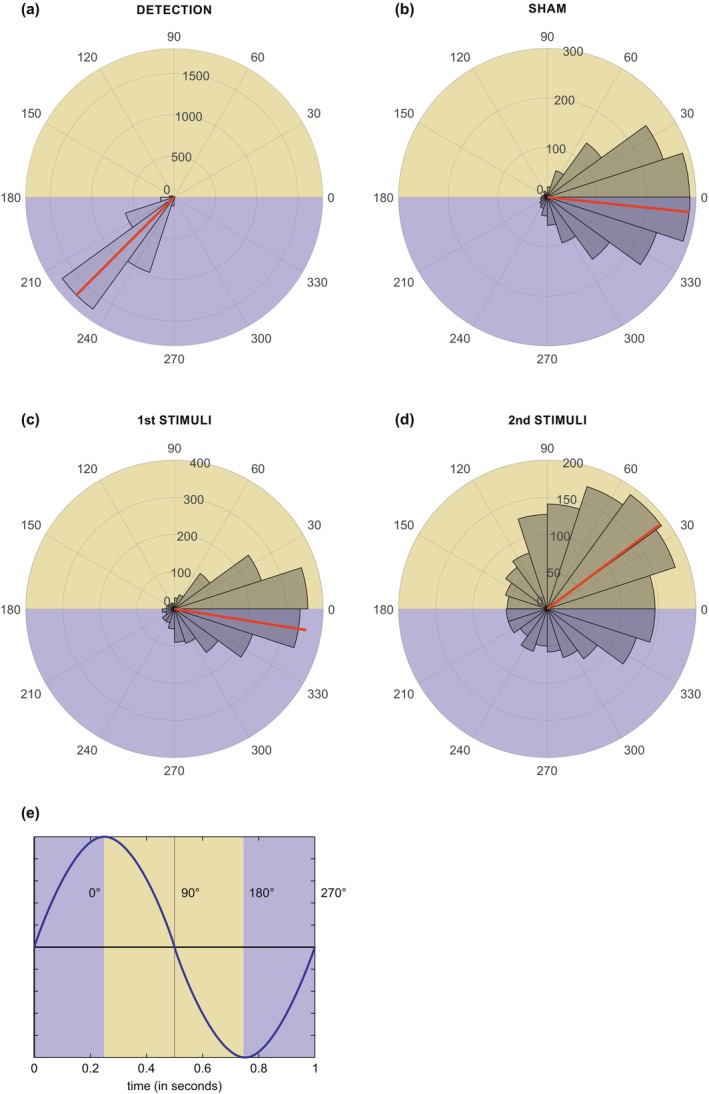
Polar histograms for detection (a), sham (b), first (c), and second (d) stimulation conditions in the NREM 3 sleep‐phase across the successfully stimulated sample (seven patients). The red line represents the mean phase value, while the grey represents the spread of phases in 30° bins across all stimulations. In sham condition (b), the stimulation phase was marked without the sound being played. (e) Graphical illustration of phase angles. The yellow and purple represent the down‐ and up‐ phase of the slow oscillations (SOs), respectively. The up‐phase of SOs was our target phase for stimulations. The sham and first stimulations were released correctly during the up‐phase; however, the second stimulations were delivered inadvertently outside of our target phase during the down‐phase of SOs.

To evaluate the accuracy of the CLAS, the polar histograms of the first and second stimulation in the SWS sleep phase show the distribution of detection and stimulation angles (Figure 1). On average, we correctly detected the SOs at 225° (SD 21°) past their trough (180°). The first sound pulse was then adequately released near the peak (360/0°) of the SOs during its up‐phase (mean [SD] 351 [50]°). The sham stimuli were also accurately timed during the up‐phase, near the peak of the SOs (mean [SD] 354 [45]°). On the other hand, the second pulse was released on average past the peak in the up‐to‐down transition of the SOs (mean [SD] 37 [66]°), which was in contrast to our primarily targeted phase.

**FIGURE 2:**
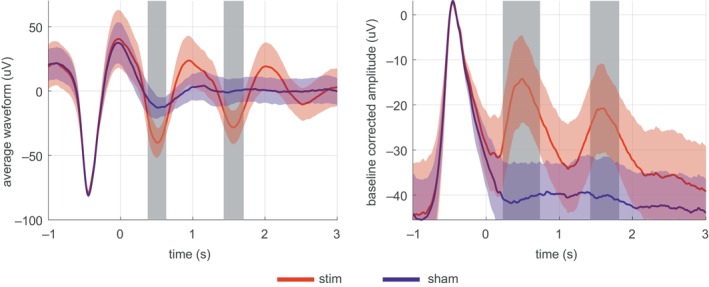
Representation of the amplitude parameter (7 patients). Zero second time point represents the presentation of the first sound stimulus. The grand average of the averaged waveform values across patients with standard deviation band around curves, for the stimulation (stim, red) and the sham (blue) conditions are shown. The corrected significant difference between stimulation and sham is marked with grey‐filled bars.

To evaluate the acute effect of CLAS, we computed the amplitude as well as relative power spectra alterations surrounding the sound stimulation events. The averaged waveform amplitude values in SWS in the stimulation and sham conditions are shown in Figure 2. The CLAS significantly enhanced the amplitude of the SO down‐phase in the next two waves following the presentation of sound stimuli.

The CLAS also significantly enhanced the mean value of relative power spectra for the SOs band (ON interval: 0.361, OFF interval: 0.354, *Z* = 3.40, *p* < 0.001). The relative power spectra in the delta band were not significantly altered after CLAS (ON interval: 0.559, OFF interval: 0.551, *Z* = 1. 740, *p* = 0.080).

### 3.3 Sleep macrostructure

Analyses of PSG recordings revealed no statistically significant differences between the stimulation and sham conditions regarding sleep macrostructure characteristics, such as sleep onset latency, TST, sleep efficiency, time and proportion of different sleep stages, and the number of arousals (Table 2). Hence, CLAS did not change any of the sleep macrostructure characteristics.

**TABLE 2:** Objective sleep macrostructure variables in the stimulation and sham night (seven patients).

Sleep variable	Stimulation, mean (± SD)	Sham, (mean (± SD)	*Z*	*p*	*r*
Sleep onset latency, min	23.93 (5.72)	24.71 (14.71)	−1.01	0.310	−0.381
Total sleep time, min	411.68 (30.28)	405.78 (58.10)	−0.16	0.866	−0.060
Sleep efficiency, %	84.33 (8.62)	83.18 (9.41)	−0.16	0.554	−0.060
Wake, min	54.96 (23.15)	60.67 (50.62)	−0.33	0.735	−0.125
Wake, %	11.69 (4.54)	12.95 (10.45)	−0.50	0.612	−0.189
Arousals, *n*	66.66 (32.94)	52.50 (18.57)	−1.18	0.237	−0.446
Arousal index, *n*/h	9.37 (4.44)	8.23 (3.12)	−0.52	0.600	−0.197
NREM 1, min	12.71 (9.26)	12.42 (6.60)	−0.17	0.865	−0.064
NREM 1, %	2.69 (1.92)	2.67 (1.30)	−0.16	0.866	−0.060
NREM 2, min	217.85 (44.60)	203.14 (39.54)	−0.76	0.446	−0.287
NREM 2, %	46.47 (8.05)	43.69 (8.34)	−0.67	0.499	−0.253
NREM 3/SWS, min	93.28 (46.21)	94.71 (31.04)	−0.16	0.866	−0.060
NREM 3/SWS, %	20.29 (10.58)	20.39 (6.57)	−0.16	0.866	−0.060
REM, min	88.07 (29.32)	95.78 (37.56)	−0.42	0.672	−0.159
REM, %	18.85 (6.00)	20.41 (7.32)	−0.50	0.612	−0.189

*Note*: Wilcoxon signed‐ranks test was used.

Abbreviations: (N)REM, (non‐)rapid eye movement sleep; SD, standard deviation; SWS, sleep‐wave sleep.

### 3.4 Power spectral analysis of SWS

**FIGURE 3:**
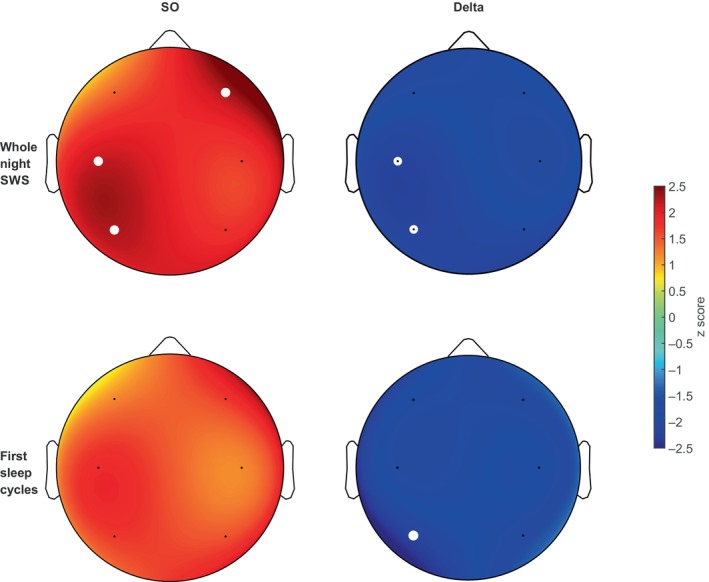
Topoplots representing increased relative slow‐wave sleep (SWS) slow oscillation SO power (red) and decreased relative SWS delta power (blue) after closed‐loop auditory stimulation across the whole night (top topoplots) and first two sleep cycles (bottom topoplots). White dots denote false discovery rate corrected significant results.

The EEG analysis of the whole‐night SWS revealed overall increases in relative SO power as well as overall decreases in relative delta power (Figure 3), suggesting that CLAS enhanced SOs, possibly at the expense of the delta power suppression. At the same time, overall absolute power remained unaltered. We also found several additional alterations of sigma activity. CLAS decreased absolute sigma power F4 (mean [SD] stimulation 0.679 [0.399], sham 0.820 [0.461]; *Z* = −2.197, *p* = 0.028) and relative sigma power at F4 (mean [SD] stimulation 0.009 [0.009], sham 0.012 [0.012]; *Z* = −2.197, *p* = 0.028).

The spectral analysis of the first two sleep cycles revealed a few alterations in sigma, SOs, and delta band after CLAS. Specifically, we observed that absolute sigma activity decreased at C3 (mean [SD] stimulation 0.610 [0.317], sham 0.736 [0.407], *Z* = −2.366, *p* = 0.018), F4 (mean [SD] stimulation 0.689 [0.391], sham 0.850 [0.465]; *Z* = −2.197, *p* = 0.028), and relative sigma power at F4 (mean [SD] stimulation 0.008 [0.008], sham 0.011 [0.009]; *Z* = −2.197, *p* = 0.028). Similarly to whole‐night SWS, absolute SOs were higher at F4 (mean [SD] stimulation 57.222 [36.692], sham 52.570 [34.950]; *Z* = −2.366, *p* = 0.018). Further significant changes in relative SOs and delta are reported in Figure 3. All significant results were FDR corrected.

### 3.5 Sleep spindle activity

During the stimulation compared to the sham night, we found a lower average amplitude of sleep spindles at C3 in SWS (mean [SD] stimulation 10.064 [2.865], sham 10.786 [3.079]; *Z* = −2.197, *p* = 0.028, corrected). CLAS did not cause any other significant changes in SWS sleep spindle characteristics including sleep spindle amplitude, density, frequency, and duration at any of the electrodes.

### 3.6 Subjective sleep quality

Subjective TST did not differ between the stimulation (mean [SD] 400.31 [67.68] min) and sham nights (mean [SD] 375 [83.42] min; *t*[15] = 1.587, *p* = 0.133, *r* = 0.138). The patients did not report feeling more rested after the stimulation night (median score = 2) or after the sham night (median score = 2; *t* = 18, *p* = 1, *r* = 0), and the results showed no difference in the subjective evaluation of sleep quality during the sham (median score = 2) and stimulation nights (median score = 2; *t* = 20, *p* = 0.405, *r* = −0.132).

### 3.7 Declarative memory

There was no significant difference in the memory task performance after the stimulation (mean [SD] −3.86 [7.56]) and sham (mean [SD] −3.25 [5.56]) condition (*Z* = 0.00, *p* = 1.000).

## 4 DISCUSSION

For the first time, the present study assessed the effect of CLAS during sleep in patients with chronic insomnia. As chronic insomnia is characterised by cortical hyperarousal (Riemann et al., 2010), possibly translating into increased vulnerability to external stimuli and decreased waking threshold (Thacher et al., 2006), we investigated whether CLAS would increase the frequency of awakenings and other signs of arousal in this clinical population. Our analyses did not reveal any difference in the number of arousals, awakenings, duration, and proportion of wakefulness nor enhanced fast EEG activity after CLAS, suggesting that CLAS does not disturb sleep in insomnia. On the contrary, the immediate effect of CLAS on SOs corresponded well with the effect previously reported in healthy volunteers (Ngo et al., 2013). The SOs showed increased amplitude following the stimuli and higher relative power in ‘ON’ (stimulation) versus ‘OFF’ intervals. Moreover, the SOs activity was enhanced throughout the whole SWS in the stimulation compared to the sham condition. Again, this is in accordance with studies on healthy participants (Ngo et al., 2013; Papalambros et al., 2017). In contrast, relative delta activity in SWS was reduced after stimulation compared to the sham, possibly favouring the SOs as the overall absolute power remained unchanged.

Our results are congruent with those of Feige et al. (2018), who found no evidence for a reduced awakening threshold in response to auditory stimuli in insomnia compared to good sleepers. In addition, a recent event‐related potentials study by the same group showed that patients with insomnia do not show altered acoustic stimuli processing in NREM sleep, although they are prone to sleep disturbance following acoustic stimuli presented in phasic REM sleep (Feige et al., 2021). In this context, CLAS delivered during deep sleep seems to be a feasible approach to insomnia. However, it is important to mention that more than half of our participants received only a small number of stimuli during sleep, which could lead to lower efficiency of the stimulation protocol. This may have been due to several factors including technical issues during recordings, e.g., temporarily worsened signal quality, or more likely factors relating to different sleep morphology in insomnia, e.g., lower power and amplitude of SOs and delta (Hogan et al., 2020; Zhao et al., 2021), lower amount of SWS, increased sleep fragmentation, and hyperarousal (Baglioni et al., 2014; Riemann et al., 2010; Svetnik et al., 2017). Although we used two amplitude thresholds for stimulation based on the age of each participant (Ngo et al., 2013; Papalambros et al., 2017), our results indicate that the threshold for stimulation needs to be further adapted and less strict to patients with insomnia to achieve sufficient opportunity for CLAS in this patient group.

Further individualisation of CLAS protocol could also be adapted for the presentation of numerous subsequent sound stimuli. In our study, the first sound pulses were delivered upon detection of a hyperpolarising slow‐wave down‐state, whereas the second sound stimuli followed after a fixed interval of 1.075 s (Ngo et al., 2013). Due to the fixed time step, the second stimulation could not take into account the variable frequency of the subsequent slow wave nor a possibly overall different characteristics of SOs in insomnia (e.g., lower amplitude or higher frequency). Our results showed that the second stimulation often occurred in the descending phase of the SO. As the down‐phase stimulation can inhibit slow waves (Ngo et al., 2013), our second stimulation might have inadvertently affected CLAS efficacy for SO enhancement as well as sleep‐dependent memory consolidation. Further studies should therefore ideally personalise the intervals between the presentation of subsequent stimuli or use only one sound stimulus to target the desired phase of the wave.

In the present study, we did not find a difference in sleep spindle density, frequency, and duration in CLAS compared to the sham condition. However, our analyses revealed lower spindle amplitude at C3 and a decreased relative sigma power after CLAS, which may point to possible suppression of spindle activity throughout the night following CLAS. As the research on spindle activity in insomnia has shown inconsistent results (Weiner & Dang‐Vu, 2016), it is difficult to draw specific conclusions about the insomnia population. Some studies showed enhanced spindle activity in insomnia, possibly suggesting an increased level of sleep‐protecting mechanisms in reaction to enhanced arousal (Spiegelhalder et al., 2012). Moreover, a fast spindle rate has been conversely associated with sleep pressure (Knoblauch et al., 2002). Reduced spindle activity in our present study could thus reflect enhanced sleep pressure due to lowered arousal after CLAS. This would be consistent with an increased SOs activity after CLAS.

Congruently with previous findings, our present study did not find any differences in sleep macrostructure (Ngo et al., 2015; Papalambros et al., 2017). TST and sleep efficiency, as well as sleep‐staging characteristics in terms of both minutes and percentage, remained unchanged. Furthermore, CLAS did not alter subjective sleep quality, which aligns with previous findings (Leminen et al., 2017; Papalambros et al., 2017). However, in the context of treating insomnia, it raises questions about whether acoustic stimulation leads to sleep changes that result in clinically meaningful improvements. Nonetheless, the current evidence indicates that CLAS does not negatively impact sleep quality. Similarly, declarative memory performance between the stimulation and sham conditions also remained unaltered. Our negative findings may be due to the limitations of our stimulation approach, which included a low number of stimulations in some participants, possibly blunting the effect of the second stimuli, and a low overall sample size. Our memory findings thus contrast with the majority of research (Wunderlin et al., 2021) although similar inconsistencies in other studies can be found (Diep et al., 2019; Schneider et al., 2020).

The present study has several limitations and provides multiple opportunities for future research. First, the present study did not include a control group, which would allow a comparison between the effects of CLAS on patients with insomnia disorder and healthy subjects using the same stimulation protocol in identical experimental conditions. A direct comparison between insomnia and healthy could bring insights into the reasons for low amount of overall stimulation in insomnia group and whether this is a problem of our used algorithm or specifically insomnia. Second, our stimulation approach was dependent on manual detection of stable SWS, which was limited by experimenters’ ability (e.g., expertise to visually detect SWS online, vigilance level, signal quality). This manual step affected the start and end of stimulation windows and thus could have influenced the amount of stimulation as well as the overall efficiency of the stimulation protocol. Third, although this study was balanced in terms of the order of the stimulation and sham conditions, providing participants with an adaptation night to eliminate the first night's effect of the sleep laboratory environment on sleep quality would be beneficial. Additionally, due to the high variability of the number of stimulations in our sample and due to the lack of knowledge of the minimum number of stimuli needed to elicit the effects of CLAS, main analyses were performed only on the patients receiving ≥50 stimuli, reducing our sample to seven patients, another limitation of this study. A relatively low success rate in the stimulated patients may be due to our SO detection algorithm being too strict for this clinical group as the character of SOs is less pronounced than in healthy controls (Hogan et al., 2020). However, further research is needed to study SOs characteristics in insomnia in more detail and to determine an optimal CLAS algorithm to see whether CLAS may be an effective approach to insomnia amelioration.

In summary, our study provides the first evidence that CLAS during sleep is feasible in chronic insomnia. This method does not increase arousal or wakefulness after sleep onset but can intervene in the sleep dynamics. In our study, the CLAS led to an immediate increase in the SOs amplitude, enhanced SOs activity, and reduced delta and sleep spindle activity in SWS across the whole night. It remains unclear whether this could be considered a sign of enhanced sleep consolidation. Further studies might benefit from adjustment of the stimulation threshold and a more precise stimulation algorithm. In addition, it is essential to explore whether>1 stimulation night would add some benefits for sleep structure and subjective sleep quality in patients with insomnia.

## AUTHOR CONTRIBUTIONS

**Daniela Dudysová:** Conceptualization; data curation; methodology; supervision; visualization; writing – original draft; writing – review and editing. **Karolina Janků:** Data curation; formal analysis; supervision; writing – original draft; writing – review and editing. **Marek Piorecký:** Methodology; software; writing – review and editing; writing – original draft; visualization; formal analysis. **Veronika Hantáková:** Investigation; methodology; writing – review and editing; project administration. **Mária Orendáčová:** Writing – review and editing; investigation; methodology; project administration. **Václava Piorecká:** Formal analysis; writing – review and editing; visualization; software. **Jan Štrobl:** Software; methodology. **Monika Kliková:** Formal analysis; writing – review and editing. **Hong‐Viet V. Ngo:** Writing – review and editing; methodology; software. **Jana Kopřivová:** Funding acquisition; conceptualization; supervision; writing – original draft; writing – review and editing.

## CONFLICT OF INTEREST STATEMENT

The authors declare that they have no conflicting interests.

## Data Availability

The data that support the findings of this study are available from the corresponding author upon reasonable request.
